# Profile of Priority Antimicrobial-Resistant Pathogens and the Behavior of Multidrug-Resistant Tuberculosis in the Santiago de Cali District, Colombia

**DOI:** 10.3390/pathogens15030329

**Published:** 2026-03-19

**Authors:** Alejandra Mondragón Quiguanas, Jorge Iván Montoya Salazar, Juan Camilo Mosquera-Hernandez, Margareth Zuluaga Aricapa, Carlos Eduardo Pinzón Flórez, German Escobar Morales, Johana Alejandra Moreno-Drada, Bruno Gutiérrez, Lucy Marcela Díaz Rivadeneira

**Affiliations:** 1 Santiago de Cali District Public Health Secretariat, Santiago de Cali Mayor’s Office, Santiago de Cali 760045, Colombia; alejandra.mondragon00@usc.edu.co (A.M.Q.); montoya.jorge@correounivalle.edu.co (J.I.M.S.); juan.mosquera4@outlook.com (J.C.M.-H.); margareth.zuluaga@cali.gov.co (M.Z.A.);; 2 Faculty of Health, School of Dentistry, Universidad del Valle, Santiago de Cali 760043, Colombia; 3 Pacifico Siglo XXI Research Group, Department of Oral Public Health, Faculty of Health, School of Dentistry, Universidad del Valle, Santiago de Cali 760043, Colombia

**Keywords:** antimicrobial resistance, drug resistance, microbial, cross-sectional studies, WHO bacterial priority pathogens, epidemiological monitoring, resistance profiles, *Enterobacteriaceae*, *Staphylococcus aureus*, *Mycobacterium tuberculosis*

## Abstract

Background: Antimicrobial resistance is a threat that increases morbidity and mortality. This cross-sectional study aimed to describe the profile of priority antimicrobial-resistant pathogens and to analyze the behavior of multidrug-resistant tuberculosis (MDR-TB) in the Santiago de Cali District, Colombia. Methods: researchers compiled information from data provided by healthcare institutions, the National Public Health Surveillance System, and laboratory-based surveillance using the World Health Organization’s WHONET v.5.6 software. Univariate statistical analysis described trends in pathogen resistance, and multivariate analysis analyzed the behavior of MDR-TB. Results: Among Gram-negative bacteria, high levels of carbapenem resistance were observed in *A. baumannii* (84% aztreonam resistance) and in *K. pneumoniae* (63%). *P. aeruginosa* exhibited elevated multidrug resistance, consistent with extensive antimicrobial selective pressure. MDR-TB exhibited a high burden of resistance, reaching 96%, with projections indicating a potential increase driven by monoresistance and resistance to rifampicin. Patients with drug-resistant tuberculosis who were HIV-positive or experiencing homelessness had a significantly higher likelihood of hospitalization (OR 5.59; 95% CI 3.09–10.11 and OR 2.94; 95% CI 1.48–5.81, respectively) and mortality (OR 3.34; 95% CI 1.72–6.49 and OR 2.59; 95% CI 1.16–5.79, respectively). Conclusions: The expansion of resistance mechanisms suggests sustained selective pressure, underscoring the need for strategies to optimize antibiotics.

## 1. Introduction

Antimicrobial resistance (AMR) is one of the greatest threats to global public health, affecting the effectiveness of medical treatments, the safety of surgical procedures, and the sustainability of health systems [1,2]. In 2019, over 1.27 million deaths were caused by antimicrobial-resistant infections, and this figure could reach ten million annual deaths by 2050 without effective intervention [3].

In 2023, one in six cases of bacterial infection were resistant [4]. In response, the World Health Organization (WHO) identified priority antimicrobial-resistant pathogens. It launched the Global Action Plan on AMR in 2015, promoting surveillance, the rational use of antimicrobials, and scientific research as fundamental pillars [5]. Since then, at least 13 new antibiotics targeting priority bacterial pathogens have been introduced and approved [6].

Following WHO recommendations, Colombia established the National AMR Response Plan (2018–2022), implemented the integrated surveillance system led by the National Institute of Health (INS), and strengthened the use of platforms such as the World Health Organization Collaborating Centre for Surveillance of Antimicrobial Resistance (WHONET) to consolidate microbiological data in public and hospital laboratories [7,8]. At the regional level, the District of Santiago de Cali has demonstrated increasing interest in AMR surveillance and analysis, given its high population density, the complexity of its hospital network, and the significant burden of infectious diseases, highlighting the importance of epidemiological surveillance of tuberculosis and other nosocomial infections caused by multidrug-resistant (MDR) bacteria. In Cali, tests based on Polymerase Chain Reaction (PCR) are the fundamental molecular methods for the rapid detection of *Mycobacterium tuberculosis* and its resistance to medications [9,10]. According to the Health Situation Analysis for the Special District of Cali in 2023, communicable and nutritional conditions accounted for 28.7% of care in early childhood (0 to 5 years), 14.8% in childhood (6–11 years), 10.3% in adolescence (12–18 years), and 13.8% in the young population (14–26 years). Of the causes of morbidity from communicable diseases, 54.7% were infectious and parasitic diseases and 39% were respiratory infections. The mortality rate for acute respiratory infections in men in the same year was 18.44 per 100,000 inhabitants, and 8.99 per 100,000 inhabitants for tuberculosis, and 1.97 in cases of sepsis. The overall incidence rate of tuberculosis in the district for 2025 is 57.24 per 100,000 inhabitants, with 85.8% attributable to pulmonary tuberculosis.

However, gaps remain in the continuous, systematic analysis of resistance patterns, especially those that integrate Gram-negative and Gram-positive bacteria and resistant mycobacteria. The inclusion of mycobacteria, particularly *Mycobacterium tuberculosis*, is especially important because resistant *M. tuberculosis* is classified as a critical priority pathogen [5].

However, gaps persist in ongoing analyses of data integration for Gram-negative, Gram-positive, and mycobacterial bacteria, and this approach is novel in Latin America, as surveillance of these groups has historically been fragmented and not integrated. AMR has focused primarily on Gram-negative and Gram-positive bacteria associated with healthcare-associated infections, using systems such as WHONET that do not routinely incorporate mycobacterial information in accordance with established guidelines. Meanwhile, mycobacteria are monitored through national tuberculosis programs, with independent systems and networks [5].

Integrating these microbial groups into a single analytical framework is therefore a novel and relevant approach in the Latin American context. This approach strengthens understanding of antimicrobial resistance dynamics in healthcare settings, optimizes clinical and programmatic decision-making, and improves strategies to prevent and control healthcare-associated infections.

This integrated approach aligns with global public health priorities. The World Health Organization included mycobacteria on its list of priority pathogens in its 2024 update, recognizing the critical threat posed by resistant strains to health systems worldwide [5]. Similarly, the 2025 Global Antimicrobial Resistance Surveillance System (GLASS) report highlights the prevalence of resistant mycobacteria in hospital settings, emphasizing the need to strengthen surveillance, analysis, and coordination with other priority bacterial groups.

Furthermore, multidrug-resistant tuberculosis (MDR-TB) is a global public health issue that, in Colombia specifically, has steadily increased since 2001, with an estimated prevalence of 13% in the most common phenotype [11,12]. The increasing resistance to antituberculosis drugs and their relationship to precarious living conditions and the presence of HIV in the affected population have impeded effective tuberculosis control [13,14]. Therefore, this study aimed to describe the profile of priority antimicrobial-resistant pathogens and analyze the behavior of multidrug-resistant tuberculosis in the Santiago de Cali District, Colombia.

## 2. Materials and Methods

### 2.1. Study Design

The researchers conducted a cross-sectional study based on microbiological data collected from January 2015 to December 2024 in the Santiago de Cali District, Colombia.

### 2.2. Data Source and Population

Researchers collected data from the Colombian National Public Health Surveillance System (SIVIGILA, Sistema de Vigilancia en Salud Pública) and the WHONET information systems between 2019 and 2024. Between 2015 and 2019, researchers collected data exclusively from SIVIGILA. They also reviewed microbiological databases generated by second- and third-level hospitals, public health laboratories, and reference centers of the National Tuberculosis Program, which were recognized in this study as healthcare provider institutions (IPS). This study included data on clinical isolates of priority bacteria classified by the WHO and the INS, including *Escherichia coli*, *Klebsiella pneumoniae*, *Pseudomonas aeruginosa*, *Acinetobacter baumannii*, *Staphylococcus aureus*, *Enterococcus faecium*, and mycobacteria of the *Mycobacterium tuberculosis* complex. Researchers excluded data from duplicate isolates from the same patient within 30 days, as well as data from environmental or incomplete samples.

The researchers analyzed microbiological data from IPS and identified microorganisms using both traditional methods (culture, Gram staining, biochemical tests) and automated systems (VITEK^®^ 2 (bioMérieux, Marcy-l’Étoile, France), BD Phoenix^®^ (Becton, Dickinson and Company, Sparks, MD, USA), and MicroScan^®^ (Beckman Coulter, Brea, CA, USA)). Similarly, they conducted Antimicrobial Susceptibility Testing (AST) in accordance with the Clinical and Laboratory Standards Institute (CLSI) guidelines for each year of the study. Specifically, CLSI M100 provided breakpoints and quality control parameters, and CLSI M45 provided guidance on susceptibility testing of antimicrobial-resistant bacterial pathogens [15,16,17].

For mycobacteria, the IPS used culture methods on solid Löwenstein-Jensen medium (Becton, Dickinson and Company, Sparks, MD, USA) and liquid (BACTEC MGIT 960 system (Becton, Dickinson and Company, Sparks, MD, USA) media, as well as phenotypic susceptibility tests for isoniazid, rifampicin, ethambutol, and pyrazinamide, according to INS and National Laboratory Network protocols [18,19].

#### Variables Collected and Data Structure

Within the analytical framework of this study, the researchers conducted all analyses using data extracted from WHONET, the standardized software officially implemented in the National AMR Surveillance System of Colombia, and the study followed the WHO Global Antimicrobial Resistance and Use Surveillance System (GLASS) methodology. The researchers structured the data at the level of individual microbiological isolates.

For each isolate, the following standardized variables were collected and analyzed:

- Microbiological identification (Bacterial or mycobacterial identification at the genus and species level; Clinical specimen type; Patient care setting (intensive care unit, general wards, or outpatient services, when available)).
- Antimicrobial susceptibility data: Antimicrobial susceptibility testing results for each antimicrobial agent, expressed as categorical interpretations (susceptible, intermediate, resistant), were automatically generated in WHONET according to the applicable CLSI breakpoints for each study year. Quantitative susceptibility data, including minimum inhibitory concentration (MIC) values and/or inhibition zone diameters, were used for internal validation and detailed phenotypic analyses. Classification of resistance phenotypes using WHONET algorithms, such as extended-spectrum β-lactamase (ESBL) production, carbapenem resistance, methicillin-resistant *Staphylococcus aureus* (MRSA), and vancomycin-resistant *Enterococcus* (VRE). Inference of clinically relevant resistance mechanisms, including carbapenemases (KPC, NDM, and OXA-48-like), based on antimicrobial susceptibility patterns and confirmation by reference laboratories when applicable.
- Epidemiological and temporal variables (Date of isolate collection (year and month); Reporting healthcare institution, coded and anonymized; Classification of clinically significant isolates according to national surveillance definitions).

The use of WHONET enabled systematic data harmonization, quality control, and integrated analyses across Gram-negative, Gram-positive, and mycobacterial species, ensuring methodological consistency, inter-institutional comparability, and alignment with national and international AMR surveillance standards.

### 2.3. Statistical Analysis

The researchers used Microsoft Excel^®^, STATA 12, WHONET version 5.6, R version 4.3, and Python version 3.11, for data consolidation, cleaning, and analysis. Univariate statistical analysis allowed for the estimation of absolute and relative frequencies to describe the distribution of sociodemographic, clinical, and microorganism variables, as well as their resistance profiles, considering isolated microorganisms, antimicrobial susceptibility profiles (susceptible, intermediate, resistant), types of resistance (MDR, extensively resistant (XDR), pan-resistant (PDR)), and year of isolation from public and private services. Similarly, they assessed temporal trends using time series analysis and compared proportions using the chi-square test for linear trend (*p* < 0.05 for statistical significance). To project the overall number of drug-resistant tuberculosis cases through 2027, the researchers employed a double Holt predictive model because the data did not exhibit stable seasonality; our primary interest was the short-to-medium term, and the model allowed for rapid adaptation to recent changes. Furthermore, the model showed a good fit and compared to simpler models, adequately captured the observed trend in the data. The researchers implemented the model in R (version 4.5.1) using the forecast package and generated the plots with ggplot2. The researchers fitted the model using the holt function. The model used a 3-period time horizon and a 95% confidence level. They evaluated the goodness of fit by estimating the mean error (ME), root mean square error (RMSE), mean absolute error (MAE), mean percentage error (MPE), mean absolute percentage error (MAPE), comparison with a naive model (MASE), and the autocorrelation of residuals (ACF1). Finally, they projected the number of cases by tuberculosis resistance types using double Holt models again (for mono-resistant, MDR, and rifampicin-resistant conditions), along with the respective goodness-of-fit tests.

Multivariate analysis to establish the association between the dependent variables (hospitalization and death of patients with drug-resistant tuberculosis) and the independent variables (homelessness, indigenous population, low socioeconomic status, and coinfection of tuberculosis and human immunodeficiency virus (HIV)) was performed using binary logistic regression to estimate the adjusted odds ratio (OR) with its corresponding 95% confidence interval. Only covariates with *p*-values less than 0.05 in the final model were statistically significant. The researchers defined the socioeconomic level variable using Colombia’s official residential classification system, the socioeconomic stratum. This system classifies households into six categories based on urban environmental characteristics. They defined socioeconomic level in three levels: low (strata 1 and 2), middle (strata 3 and 4), and high (strata 5 and 6). Similarly, the researchers classified the types of social security based on the Colombian social security system. Colombia has a mandatory public health insurance system that includes: a Contributory Regime for people who pay income-based contributions; a Subsidized Regime financed by the government for low-income populations; Special Regimes that cover specific occupational groups; and an Exception category for people covered by alternative legal systems.

## 3. Results

The results showed an exponential increase in the number of reported isolations from 2019 to 2020. During 2019, isolations across different healthcare services reached 102,427, while in 2020 they registered the highest number recorded during the period, with 838,826 isolations. In 2021, the findings show a notable decrease, with 106,156 isolations reported. However, from 2022 to 2024, an upward trend reappeared, though on a smaller scale: 89,488 isolations were reported in 2023 and 113,043 in 2024.

### 3.1. Distribution of Microorganisms by Service

**Figure 1:**
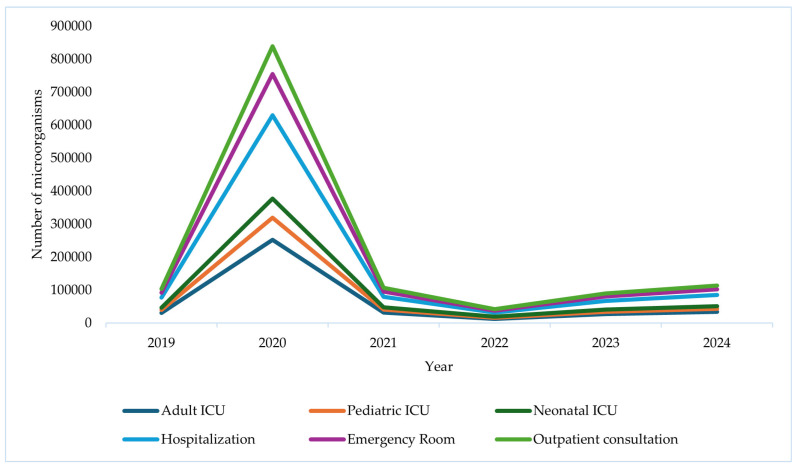
Microorganism frequency by service, Cali, 2019–2024. Source: WHONET Surveillance, Santiago de Cali, cut-off date: 2024.

There was a noticeable increase in microorganisms in 2020 across all services, especially in the adult intensive care unit (ICU) and inpatient wards. This increase coincided with increased hospitalizations and intensive antimicrobial use during the SARS-CoV-2 pandemic (from March 2020 to July 2022). This information indicates that resistance trends change during the SARS-CoV-2 peak. In 2021, the findings revealed a sharp decline in isolates, likely associated with fewer severe cases and the gradual return to normal hospital operations. Starting in 2022, the trend showed more stable values, with a slight increase in 2023 and 2024. The adult ICU and inpatient wards consistently accounted for the highest proportion of microorganisms, while the pediatric ICU, neonatal ICU, emergency department, and outpatient clinic recorded lower numbers (Figure 1).

A marked increase in the isolation of microorganisms was observed during the SARS-CoV-2 pandemic, particularly in 2020, coinciding with a period of high hospital demand. This increase was mainly concentrated in adult intensive care units and hospitalization services, reflecting the surge in admissions of critically ill patients, prolonged hospital stays, and the expanded use of invasive medical devices. The elevated volume of microbiological isolates during this period is consistent with the intensified healthcare activity and increased diagnostic samp6564_ling associated with the management of SARS-CoV-2 patients. In contrast, a subsequent decrease in isolations was observed in 2021 and 2022, followed by a gradual upward trend in the post-pandemic period, in parallel with the progressive normalization of healthcare services.

### 3.2. Distribution of the Main Microorganisms

**Figure 2:**
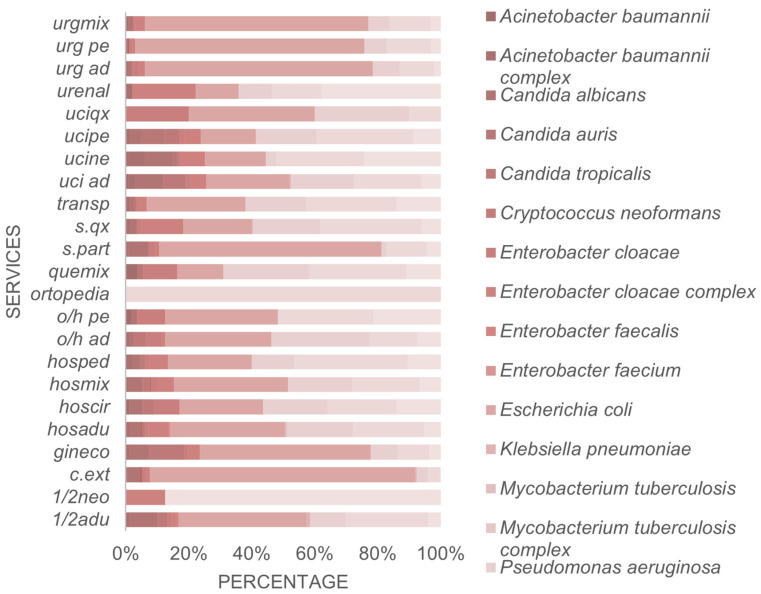
Proportion of microorganisms per service, Cali, 2019–2024. Source: WHONET Surveillance, Santiago de Cali, cut-off date: 2024. Services: 1. Inpatient Care: Intermediate Services (o/h ad), Mixed Inpatient (hosmix), Adult Inpatient (hosadu), and Adult Intermediate Stay Inpatient (1/2adu). 2. Intensive Care Units: Surgical Intensive Care Unit (uciqx) and Neonatal Intensive Care Unit (ucine). 3. Emergency Services: Mixed Emergency Department (urgmix) and Adult Emergency Department (urg ad). 4. Surgical Services: s.qx. 5. Outpatient Clinic: c.ext. 6. Patient Transport (transp). 7. Delivery Room (s.part) and 8. Orthopedics.

*Klebsiella pneumoniae*, *Escherichia coli*, and *Staphylococcus aureus* are more common across medical services, especially in inpatient wards and intensive care units. *Pseudomonas aeruginosa* and *Acinetobacter baumannii* are also prominent in critical care settings, such as the ICU and emergency departments, reflecting their roles in infections associated with invasive devices. *Candida albicans*, *Candidozyma auris*, and *Candida tropicalis* are more prevalent in ICUs and surgical services, suggesting their association with immunocompromised patients or the use of broad-spectrum antibiotics. *Enterococcus* spp. (*faecalis* and *faecium*) isolates are significantly distributed in inpatient and orthopedic wards, while *M. tuberculosis* and its complex are more frequent in outpatient clinics and some hospital services (Figure 2). Overall, the most complex services exhibit the most incredible diversity and frequency of microorganisms.

During 2019–2024, a total of 1,291,758 isolates were recorded, with the majority from hospitalized patients. Grouping the inpatient services (adult ICU, pediatric ICU, neonatal ICU, and general hospitalization), the researchers identified 968,816 isolates, concentrated mainly in the adult ICU and general hospitalization (387,527 each), followed by the pediatric ICU (103,339) and the neonatal ICU (90,423). In contrast, non-hospitalized patients, seen in the emergency department and outpatient clinics, contributed 322,940 isolates, with the emergency department (193,763) contributing more than outpatient clinics (129,177). This detailed quantification highlights the predominance of isolations in the hospital setting. It enables a more precise understanding of the relative burden between hospitalized and non-hospitalized patients, thereby complementing and expanding the information presented in Figure 2.

#### Distribution of Isolates by Body Site and Year, Cali, 2019–2024

During 2019–2024, the distribution of microbiological isolates in Cali showed a consistent predominance of urinary tract samples, which accounted for approximately one-third of all isolates each year. Bloodstream isolates were the second most frequent source, reflecting the ongoing burden of bacteremia and infections associated with intravascular devices. Respiratory tract samples accounted for a substantial proportion of isolates, particularly in 2020, coinciding with increased hospitalizations and respiratory infections during the SARS-CoV-2 pandemic. Isolates from surgical wounds and soft tissues remained relatively stable over the study period, while those associated with invasive devices increased notably in 2020 and gradually stabilized in subsequent years. Overall, these findings underscore the continued importance of urinary, bloodstream, and device-associated infections as priority targets for infection prevention and control in healthcare settings. Table 1 describes this distribution.

### 3.3. Types of Carbapenemases in ESKAPE-Like Microorganisms

**Figure A1:**
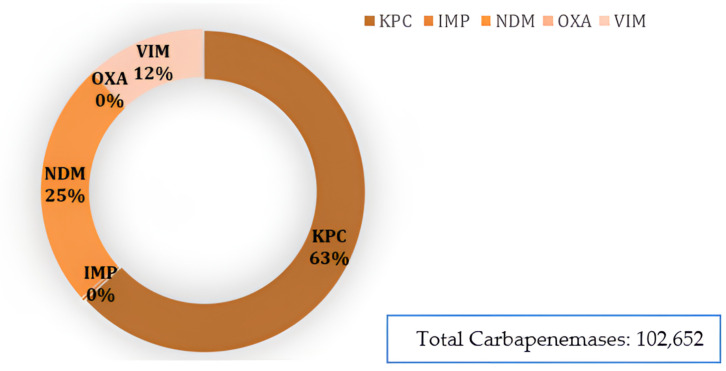
Proportion of carbapenemases in ESPKAPE agents, Cali 2019–2024. Source: WHONET, Cali Public Health Secretariat. Carbapenemases: KPC: *Klebsiella pneumoniae* carbapenemase, NDM: New Delhi metallo-β-lactamase, VIM: Verona integron-encoded metallo-β-lactamase, IMP: Imipenemase metallo-β-lactamase, and OXA: Oxacillinase carbapenemase.

An analysis of carbapenemase types in ESKAPE-type microorganisms in Santiago de Cali from 2019 to 2024 shows that carbapenemase-producing *Klebsiella pneumoniae* (KPC) is the predominant enzyme, representing 63% of isolates, followed by New Delhi metallo-β-lactamase (NDM) at 25% and Verona integron-encoded metallo-β-lactamase (VIM) at 12%. Imipenemase metallo-β-lactamase (IMP) and Oxacillinase carbapenemase (OXA) were not detected in any isolates (Figure A1). The high prevalence of KPC reflects the persistence of KPC-type carbapenemase-producing *Enterobacteriaceae*, especially *Klebsiella pneumoniae*, associated with hospital-acquired infections in high-complexity settings. The detection of NDM and VIM, although less frequent, indicates the circulation of metallo-β-lactamases in the region, which is concerning given their broad-spectrum resistance and the therapeutic limitations they entail.

### 3.4. Antimicrobial Resistance Profile in the Santiago de Cali District

**Figure 3:**
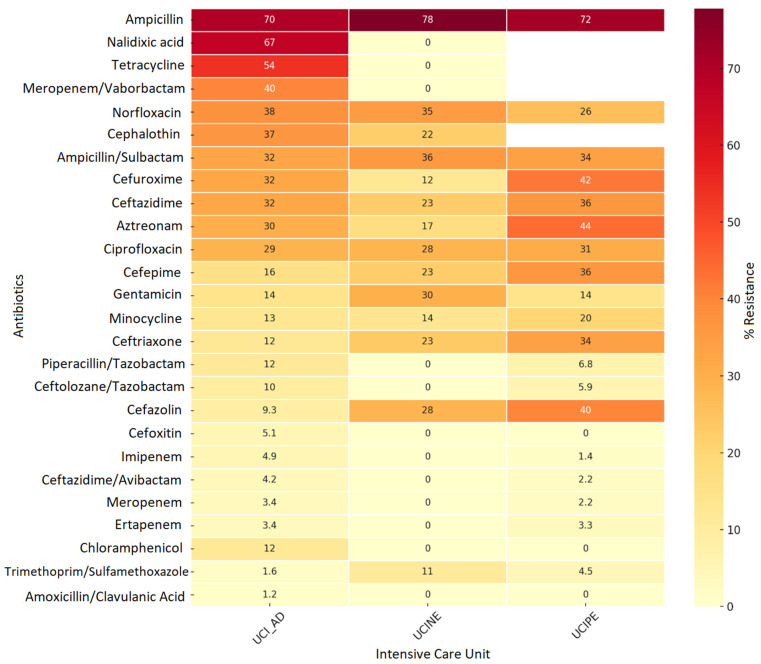
Resistance Profile of Enterobacterial Microorganisms in Intensive Care Units, Cali, 2019–2024. Source: WHONET Surveillance, Santiago de Cali, cut-off date: 2024. Services: 1. UCI_AD: Adult Intensive Care Unit. 2. UCINE: Neonatal Intensive Care Units. 3. UCIPE: Pediatric Intensive Care Unit.

Analysis of antimicrobial resistance in *Enterobacteriaceae* isolated from adult, pediatric, and neonatal ICUs between 2019 and 2024 revealed ampicillin resistance rates of up to 83.2% in adult ICUs and 75% in pediatric ICUs, highlighting the ineffectiveness of this antibiotic against most *Enterobacteriaceae* (Figure 3). The findings also revealed high levels of resistance to third-generation cephalosporins, including ceftazidime, cefepime, and cefuroxime, with rates of 30–59%, suggesting a high prevalence of extended-spectrum beta-lactamases (ESBLs). Resistance to carbapenems, such as meropenem and imipenem, remained below 30%, although their presence in pediatric ICUs is noteworthy given their restricted use. Aminoglycosides (amikacin and gentamicin) and last-line antimicrobials (colistin and polymyxin B) exhibit low resistance rates (<10%) and remain rescue therapy options (Figure 3). This situation reflects the selective pressure generated using β-lactams and fluoroquinolones, which show moderate resistance (30–43%). It emphasizes the importance of strengthening antimicrobial stewardship programs (ASPs) and microbiological surveillance to prevent the spread of resistance mechanisms, especially in critical care environments.

The distribution of carbapenemases is consistent with resistance profiles previously observed in Enterobacterales, where high rates of resistance to β-lactams, especially ampicillin and third- and fourth-generation cephalosporins, have been identified. These findings are compatible with the production of extended-spectrum β-lactamases and KPC-type carbapenemases. Likewise, the presence of NDM and VIM is associated with observed carbapenem resistance (10–28%), as these enzymes provide broad-spectrum resistance to β-lactams, including carbapenems. Overall, the findings present a resistance landscape dominated by class A carbapenemases and metallo-β-lactamases (class B). On the other hand, the absence of IMP and OXA detection suggests that the spread of these mechanisms remains limited in the population studied.

**Figure 4:**
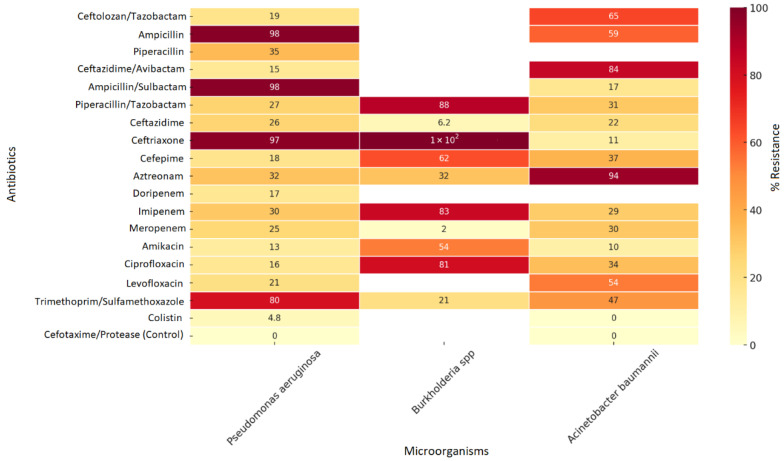
Resistance Profile of Non-Fermenting Microorganisms in Hospital Services, Cali, 2019–2024. Source: WHONET Surveillance, Santiago de Cali, cut-off date: 2024.

When evaluating the resistance profile of non-fermenting microorganisms, researchers observed high resistance levels to β-lactams such as ampicillin (98%), ampicillin/sulbactam (98%), and ceftriaxone (97%), as well as to trimethoprim/sulfamethoxazole (80%) in *Pseudomonas aeruginosa*. However, the researchers found lower resistance rates against colistin (4.8%) and ceftazidime/avibactam (15%), positioning them as last-line therapeutic alternatives. Similarly, they noted high resistance in *Burkholderia* spp. to piperacillin/tazobactam (88%), ciprofloxacin (81%), and meropenem (83%), highlighting the therapeutic challenges posed by this multidrug-resistant pathogen. For its part, *Acinetobacter baumannii* shows a critical resistance profile, with high resistance to aztreonam (84%) and intermediate resistance to fluoroquinolones and aminoglycosides (30–54%) (Figure 4).

Overall, *Acinetobacter baumannii* and *Burkholderia* spp. exhibit high levels of multidrug and carbapenemase resistance, whereas *Pseudomonas aeruginosa* offers more effective treatment options with colistin, aminoglycosides, and combinations of β-lactams with new-generation β-lactamase inhibitors (Figure 4).

**Figure A2:**
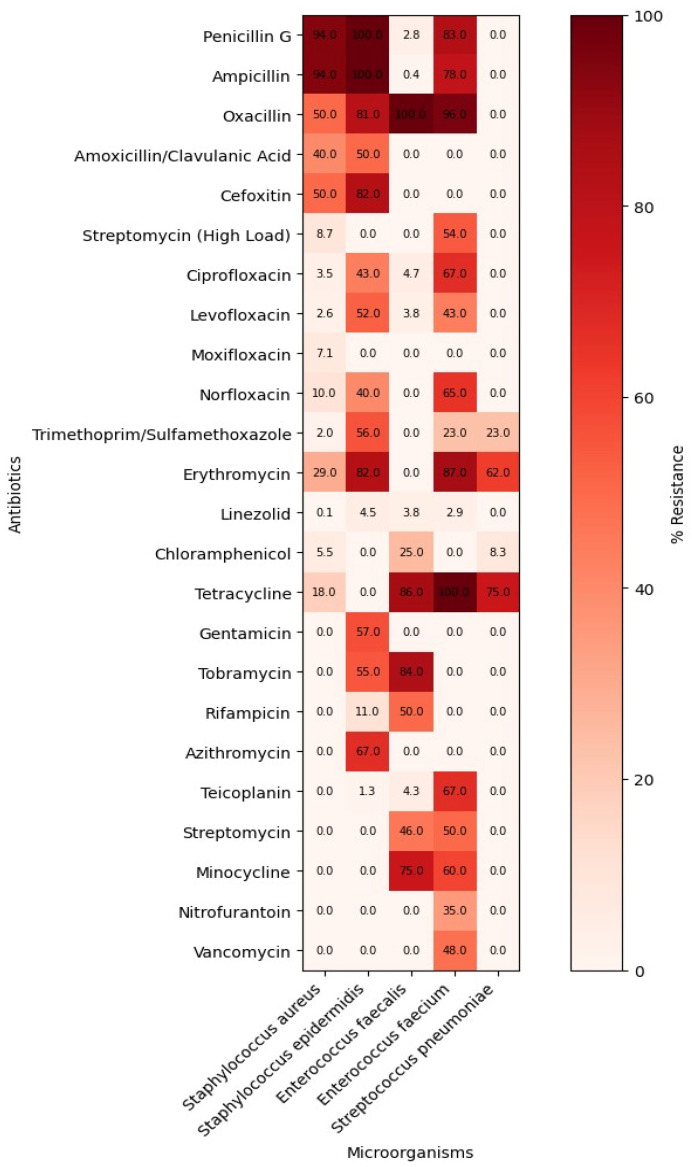
Resistance Profile of Gram-positive Microorganisms in hospital services, Cali, 2019–2024.

*Staphylococcus aureus* exhibited high resistance rates to traditional β-lactams such as penicillin G (94.1%) and ampicillin (94.3%), with concerning levels of resistance to oxacillin (50.4%) and cefoxitin (49.8%) as well, suggesting a significant presence of methicillin-resistant strains (MRSA). However, it showed good sensitivity to antibiotics such as linezolid (0.1%), trimethoprim/sulfamethoxazole (2%), and fluoroquinolones (ciprofloxacin and levofloxacin), with resistance rates under 5%. *Staphylococcus epidermidis* displayed a more complex resistance profile, with resistance rates exceeding 80% for penicillin G, ampicillin, oxacillin, cefoxitin, and erythromycin, indicating a multidrug-resistant phenotype. Although linezolid (4.5%) and teicoplanin (1.3%) remained effective, the researchers observed resistance to fluoroquinolones, with levofloxacin resistance reaching 52.3%. *Enterococcus faecalis* maintained good susceptibility to most antibiotics assessed, except oxacillin (100%) and high-dose aminoglycosides such as tobramycin (84%). It exhibited low resistance to linezolid (3.8%) and teicoplanin (4.3%), as well as high sensitivity to β-lactams such as ampicillin (0.4%) and penicillin G (2.8%). In contrast, *Enterococcus faecium* demonstrated a broader resistance pattern, with high resistance to β-lactams, fluoroquinolones (ciprofloxacin 66.7%), and erythromycin (86.9%), as well as 48% resistance to vancomycin, raising concern about the presence of VRE (vancomycin-resistant enterococci) strains. Although linezolid (2.9%) remained effective, therapeutic options became more limited. Finally, *Streptococcus pneumoniae* exhibited high resistance to tetracycline (75%) and erythromycin (62.5%), while maintaining good sensitivity to chloramphenicol (8.3%) and moderate sensitivity to trimethoprim/sulfamethoxazole (23.1%) (Figure A2).

**Figure 5:**
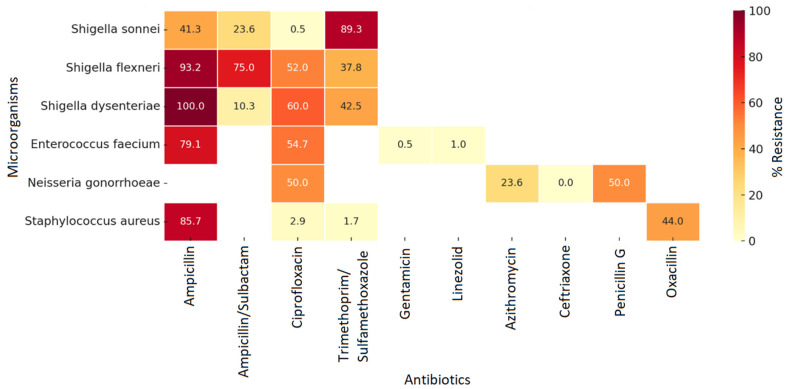
Resistance profile in other high-priority microorganisms in hospital services, Cali, 2019–2024. Source: WHONET Surveillance, Santiago de Cali, cut-off date: 2024.

*Shigella dysenteriae* showed remarkably high resistance to ampicillin (100%) and ciprofloxacin (60%), while *Shigella flexneri* exhibited similarly elevated levels of resistance to ampicillin (93.2%) and ampicillin/sulbactam (75%). *Enterococcus faecium* demonstrated significant resistance to ampicillin (79.1%) and ciprofloxacin (54.7%), while maintaining low resistance to gentamicin and linezolid. *Staphylococcus aureus*, on the other hand, remained susceptible to most of the antimicrobials assessed, although it showed concerning resistance rates to ampicillin (85.7%) and oxacillin (44%). Finally, *Neisseria gonorrhoeae* showed 50% resistance to penicillin G and ciprofloxacin, with no evidence of resistance to cefixime or ceftriaxone in the data analyzed (Figure 5).

### 3.5. Behavior of Drug-Resistant Tuberculous Mycobacteria in the Cali District 2015–2024

**Figure A3:**
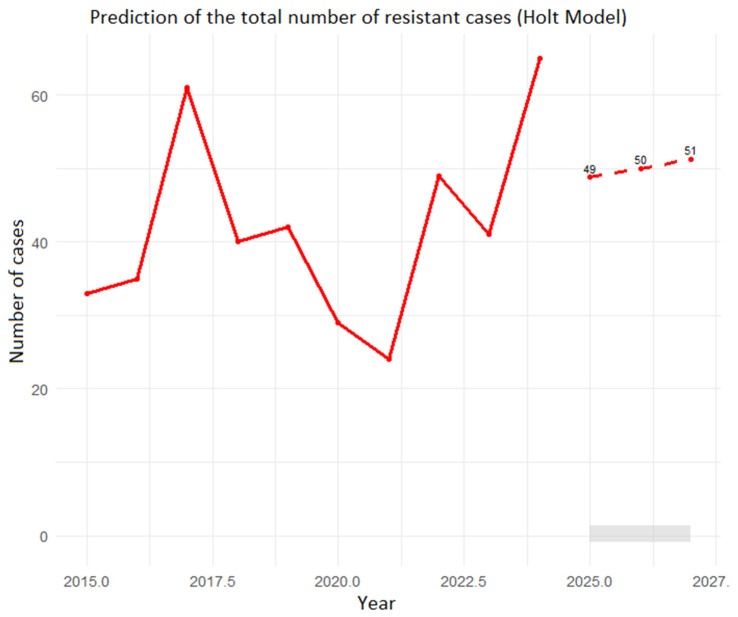
Distribution and projection of cases of drug-resistant tuberculosis in Cali, 2015–2027. Source: SIVIGILA, cut-off date 4 April 2025.

Over the past nine years (2015–2024) in the Santiago de Cali district, the public health surveillance system has recorded 419 patients with drug-resistant tuberculosis. On average, forty-two cases of drug-resistant tuberculosis are reported each year, with an upward trend in the number of cases in the city. In 2024, the area experienced the highest number of cases (65 patients). Using a three-year double-exponential Holt model, the analysis predicts that forty-nine people will be diagnosed in 2025 (95% CI: 18–79), fifty people in 2026 (95% CI: 20–80), and fifty-one people in 2027 (95% CI: 21–82) (Figure A3).

Although the model has wide prediction intervals, and therefore estimates should be interpreted with caution, it demonstrated a good fit (MASE < 1, independent residuals) and anticipates a moderate increase over the three years. The mean errors (ME ± 0 and the MPE −8.8%) indicate no significant systematic bias, while the MAPE of 23% reflects acceptable accuracy. The MASE of 0.64 and the ACF1 ± 0 support the adequacy of the fit. See Figure A3.

According to official figures from the National Administrative Department of Statistics (DANE), the population of Cali in 2024 was 2,283,846; of these, 2,483,407 were insured in the health system, of whom 1,513,605 were in the contributory regime, 934,397 in the subsidized regime, and 35,405 in the special regime. In general, women, people aged 29–59, individuals with low socioeconomic status, individuals of mixed ethnicity, and individuals enrolled in the subsidized health insurance program accounted for the largest share of reported cases. When describing the type of resistance in patients diagnosed with tuberculosis, researchers noted that over the past nine years, mono-resistant and multidrug-resistant tuberculosis represented most cases in the city (44% and 29%, respectively) (Table 2).

In 2024, drug-resistant tuberculosis cases in Cali were most common among men (66%), individuals enrolled in the subsidized healthcare system (49%), those aged 29–59 years (57%), and people of mixed ethnicity (94%). The pulmonary form accounted for most cases (96%). Regarding the resistance pattern, monoresistance was the most prevalent (37%), followed by rifampicin resistance (31%).

**Figure 6:**
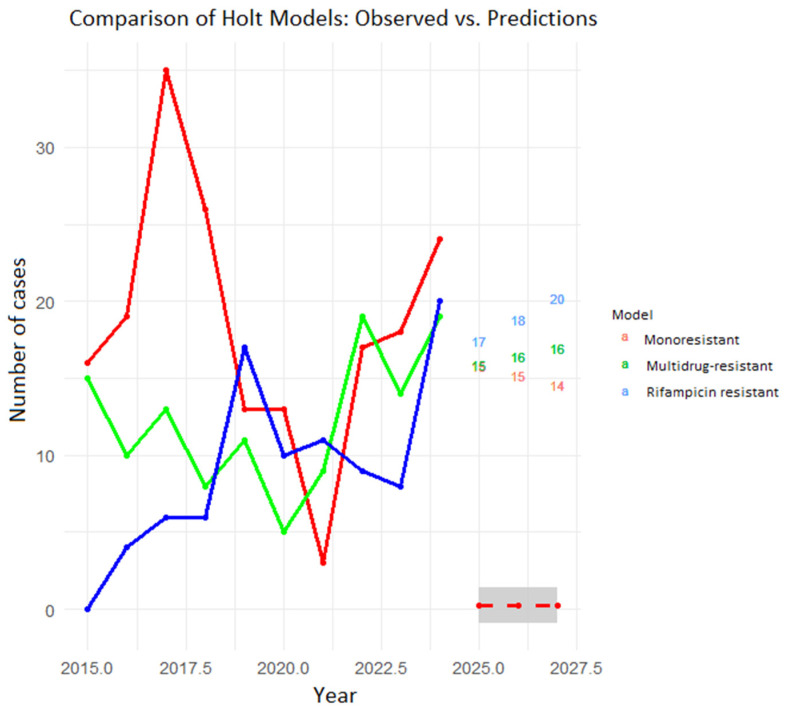
Behavior and projection of cases by resistance type in drug-resistant tuberculosis, Cali, 2015–2027. Source: SIVIGILA, cut-off date 4 April 2025.

Analyzing trends and projections by tuberculosis resistance type in Cali using double Holt models (for mono-resistant, MDR, and rifampicin-resistant conditions), the predictions indicate that rifampicin-resistant and MDR conditions will increase during the period 2025–2027. The MDR prediction for 2025 is 15 cases (95% CI: 5–26); for 2026, 16 cases (95% CI: 5–26); and for 2027, 16 cases (95% CI: 6–27). Rifampicin-resistant conditions showed a greater upward trend. The prediction indicates that by the year 2025, there will be 17 cases (95% CI 7–27); by the year 2026, 18 cases (95% CI 8–28); and by the year 2027, 20 cases (95% CI 10–29) (Figure 6).

In the model fit assessment, the researchers found that both models showed no significant bias (ME ± 0), low absolute errors (RMSE and MAE), nearly random residuals (ACF1 ± 0), and outperformed a naive model (MASE < 1). These results indicate robust, dependable predictive models.

In a multivariate analysis of data collected by the drug-resistant tuberculosis surveillance system from 2015 to 2024, which included 419 records of patients with drug-resistant tuberculosis, 66 patients with HIV, and 42 homeless people were reported for that observation period, in addition to 163 hospitalized patients and 50 deaths, researchers found that patients diagnosed with drug-resistant tuberculosis and HIV coinfection were 5.5 times more likely to be hospitalized than those with drug-resistant tuberculosis without HIV coinfection. Furthermore, the likelihood of hospitalization for homeless individuals diagnosed with drug-resistant tuberculosis was nearly three times higher than for individuals who tested positive for drug-resistant tuberculosis but were not homeless. The pseudo-R^2^ for the hospitalization model was 0.0838.

Consequently, in the city, the probability of death for patients diagnosed with drug-resistant tuberculosis and HIV coinfection is three times higher than for those with this tuberculosis condition without HIV coinfection. Similarly, homeless individuals with drug-resistant tuberculosis have a higher mortality rate (2.5 times) compared to those who are not part of this group (Table 3). The pseudo-R^2^ of the model was 0.0544.

**Figure 7:**
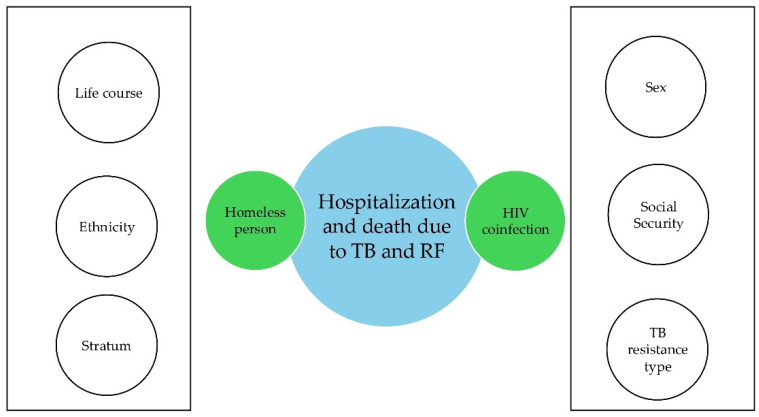
Acyclic diagram targeting drug-resistant tuberculosis Cali, 2015–2024.

Although HIV coinfection and homelessness were the only statistically significant independent variables for hospitalization and death outcomes among patients with drug-resistant tuberculosis, the researchers included other variables in the analysis because of their potential confounding effects. These included life course, sex, type of social security coverage, ethnicity, socioeconomic status, and type of resistance. These variables were directly related to the outcome variables and were therefore considered confounding factors. A directed acyclic plot is shown below, in which the statistically significant independent variables intersect the response variables. The white circles at the ends represent the confounding variables considered in the study (Figure 7).

## 4. Discussion

The results of this study show a sustained and concerning increase in antimicrobial resistance among priority pathogens in the Santiago de Cali District from 2015 to 2024, with a peak in 2020. This trend may partly stem from increased antimicrobial use during the SARS-CoV-2 pandemic, associated with both self-medication and empirical antibiotic use in hospitalized patients, which promoted selective pressure and favored the development of resistant strains [7,8]. The further increase observed from 2022 to 2024 suggests the persistence of structural determinants, such as inappropriate prescribing in outpatient settings, self-medication, and limitations in the regulation of antimicrobial rational use [20,21]. These findings are consistent with trends reported by the RENAVIGILA and WHO’s GLASS, which warn of a global increase in multidrug-resistant isolates, especially in high-complexity hospitals [4,22,23].

A key finding is the persistence of KPC-producing *Klebsiella pneumoniae* and ESBL-producing *Escherichia coli*, which the WHO classifies as critical threats [5]. The spread of these resistance mechanisms indicates ongoing selective pressure, associated with the empirical use of broad-spectrum antibiotics, gaps in infection control programs, and weaknesses in ASPs.

According to Betancur et al. [24], Carbapenemase-producing pathogens are endemic in Latin America. MDR Gram-negative pathogens harbor a wide range of carbapenemases, particularly among *Enterobacterales* and the non-fermenting bacilli *Pseudomonas* spp. and *Acinetobacter* spp. [24].

This pattern has also been observed in other Colombian cities, such as Bogotá, Medellín, and Barranquilla, underscoring the need for a stronger, coordinated national response [23,24].

According to De la Cadena et al. [25], Colombia exhibited a high rate of endemic KPC circulation since the mid-2010s, associated with both the clonal expansion of high-risk lineages and the horizontal dissemination of plasmids carrying the *bla*KPC gene [same citation as De la Cadena]. Several factors could explain this predominance. First, the high selective pressure exerted by the intensive use of carbapenems and broad-spectrum cephalosporins, especially in adult intensive care and hospital wards, favors the persistence and spread of KPC-producing strains [26].

This situation has been highlighted by Pallares et al. [27], who first describe the gaps in the implementation and effective monitoring of Antimicrobial Stewardship Programs (ASPs), including prolonged empirical prescribing, unnecessary escalation, and delayed de-escalation [27]. Second, hospital circulation and cross-transmission play a key role. The recurrent detection of KPC across multiple institutions suggests not only sporadic introductions but also sustained transmission, facilitated by limitations in infection prevention and control measures, including suboptimal adherence to hand hygiene, contact isolation, and active microbiological surveillance.

When comparing these findings with those from other countries in the region, a significant contrast emerges while KPC predominates in Colombia, Brazil, and Argentina [28], different areas of Latin America have reported a progressive increase in NDM or OXA-48-like carbapenemases. This difference suggests that, in addition to global factors, local antibiotic use dynamics, regulatory policies, and microbiological surveillance capacity decisively influence the selection of the predominant mechanism. In the city of Santiago de Cali, Colombia, the persistence of KPC could reflect a combination of established endemicity, sustained antimicrobial pressure, and limited detection of other emerging mechanisms [29], particularly in contexts where molecular characterization is not systematic.

Furthermore, researchers observed a progressive increase in carbapenem resistance among *Pseudomonas aeruginosa* and *Acinetobacter baumannii*, which are considered last-line antibiotics. These findings are consistent with the literature and pose a significant risk in intensive care units, where these pathogens are common, and treatment options are limited [30,31]. The emergence of XDR and PDR strains in some hospitals within the district, although isolated, reinforces the urgent need to strengthen containment measures, active surveillance, and environmental disinfection [32,33].

On the other hand, progressive reductions in sensitivity to glycopeptides and oxazolidinones were documented in methicillin-resistant *Staphylococcus aureus* (MRSA) strains, especially in community isolates. This phenomenon highlights changes in transmission patterns and the importance of surveillance in hospitals and the community.

In 2024, the incidence of drug-resistant tuberculosis in Cali was 1.6 cases per 100,000 inhabitants, higher than the national rate (1.02 cases per 100,000 inhabitants). These findings are consistent with the data reported by the National Institute of Health (INS) in 2024 at the national level for sociodemographic variables, noting that most patients with drug-resistant tuberculosis were male (66% vs. 66.3%), enrolled in the subsidized health insurance system (49% vs. 54.1%, respectively), and had pulmonary tuberculosis more frequently (96% vs. 93.6%). Additionally, in 2023, the type of resistance reported was similar at both the national and local levels, with monoresistance being the most frequent (46.4% and 42%), followed by multidrug-resistant tuberculosis (MDR) (25% and 40%), and rifampicin resistance at the national level of 24.8% and in the Cali district of 17% [23,29].

Tuberculosis resistance is increasing. A 2010 study by Rojas et al. found primary resistance to one of the drugs assessed in 7.6% of *Mycobacterium tuberculosis* isolates [34]. Another research by Puerto et al., published in 2023, showed that between 2013 and 2018, out of 80,601 tuberculosis cases in the country, 0.74% were multidrug-resistant tuberculosis (MDR-TB) (597 cases), with resistance accounting for 17.42% of the total in 2018, a lower rate than reported in this study for 2024. Valle del Cauca was the second department with the most cases, especially among incarcerated individuals who had received prior treatment (59.8%), similar to this study, in which prior treatment was present in 58% of cases. Additionally, malnutrition (19.43%, *p* = 0.033), diabetes (12.9%), and HIV coinfection (12.23%, *p* < 0.0001) were the most common comorbidities, similar to this study (38%, 11%, and 22%, respectively). The findings from this study showed that HIV coinfection in patients with drug-resistant tuberculosis increased the likelihood (up to 5.5 times) of hospitalization [35]. This information matches a 2010 study in which HIV coinfection with MDR tuberculosis in previously treated cases was 25% [36].

In Latin America and the Caribbean, the estimated prevalence of multidrug-resistant tuberculosis (MDR-TB) and rifampicin-resistant tuberculosis (RFTB) has been reported as 0.15% and 0.05%, respectively, in the general population, and as 0.12% and 0.03%, respectively, among people coinfected with HIV [37]. Regarding mortality, a multicenter cohort study including 582 patients from Africa, Latin America, and Asia reported a higher overall mortality rate in patients with drug resistance than in those susceptible to antituberculosis drugs (19% vs. 6%), with similar mortality rates for MDR-TB (16%) and rifampicin-resistant TB (17%). When individuals were coinfected with HIV, mortality increased to 29% and 38%, respectively [31]. Furthermore, the study by Alemu A. et al., published in 2021, showed that comorbidities such as diabetes (HR = 2.05, 95% CI 1.40–2.70), a history of previous tuberculosis (HR = 1.46, 95% CI 1.19–1.72), and HIV coinfection (HR = 2.35, 95% CI 1.68–2.82) were predictors of mortality in patients with MDR/rifampicin-resistant tuberculosis. Similarly, in this study, 6% of patients died, and the presence of HIV coinfection in drug-resistant tuberculosis increased the risk of death up to three times compared to those without HIV coinfection [38]. These findings emphasize the importance of prioritizing early diagnosis, intensified treatment, and follow-up in these patients to reduce the risk of fatal outcomes.

In accordance with WHO guidelines and national policies, since the introduction of molecular tests such as GeneXpert for diagnosing infections, efforts are underway to use it as a primary diagnostic strategy, increasing the rate of early diagnosis and early detection of resistance genes, enabling timely adjustments to appropriate treatments [39,40,41,42]. In this study, molecular testing currently available in the country was used to diagnose 87% of cases.

Drug-resistant tuberculosis, including MDR and rifampicin-resistant types, has shown a yearly decrease in cases according to WHO data. In 2015, MDR/rifampicin-resistant tuberculosis made up 4.1% of new cases and 20% of previously treated cases, dropping to 3.2% and 16%, respectively, by 2023 [43]. However, this issue still presents a significant public health challenge because treatment success rates are low (under 70% worldwide and under 60% nationally), and the high mortality rate associated with MDR can reach up to 21% [37,38,44]. This situation challenges the achievement of the Sustainable Development Goals and the End TB strategy [45,46], as evidenced by a 2024 study in The Lancet that reports, despite recent progress in lowering the global tuberculosis burden, the first provisional milestones of the WHO’s End TB strategy for 2020 were not achieved [47].

Additionally, the mortality burden of tuberculosis caused by antimicrobial resistance from 1990 to 2021 places it among the top 10 bacterial pathogens, with approximately 36,400 deaths attributable to multidrug resistance, ranking below methicillin-resistant *Staphylococcus aureus*, *Acinetobacter baumannii*, *Pseudomonas aeruginosa*, and carbapenem-resistant *Klebsiella pneumoniae* [38,48]. Considering the high prevalence of tuberculosis due to its airborne transmission, challenges in accessing diagnosis and treatment, and the non-fatal health burden it causes, the WHO included tuberculosis in its list of priority bacterial pathogens in the critical group in 2024, alongside carbapenem-resistant *Acinetobacter baumannii* and Enterobacterales resistant to carbapenems and third-generation cephalosporins [5,6].

On the other hand, it is relevant to note that mortality among patients with MDR and rifampicin-resistant tuberculosis is also associated with low treatment adherence, which was approximately 68% globally and around 57% in Colombia for 2021. Low adherence is related to the administration of multiple drugs, treatment duration, and adverse effects [11,44]. In addition, Varela L. et al. in 2023 found that unsuccessful treatment for tuberculosis is independently associated with a history of homelessness (ORa = 2.45; 95% CI 1.54; 3.89), drug dependence (ORa = 1.95; 95% CI 1.24; 3.03), previous incomplete treatment (ORa = 2.34; 95% CI 1.62; 3.38), coinfection with tuberculosis/HIV (ORa = 1.69; 95% CI 1.00; 2.86) or diabetes (ORa = 1.89; 95% CI 1.29; 2.77) [44].

Furthermore, it is crucial to continue implementing and strengthening the strategies outlined in the Global Action Plan on AMR [49,50]. Regarding antimicrobial resistance associated with tuberculosis, it is crucial to strengthen disease control programs by adopting public policies that ensure equitable, rapid, and timely access to diagnostic tests and treatments. These actions should focus on populations most vulnerable to disease and to fatal outcomes. In addition, it is vital to highlight the use of more accurate and faster diagnostic tools, such as current molecular tests, and to develop strategies to detect antimicrobial resistance in M. tuberculosis to new drugs, such as bedaquiline [51]. Likewise, it is essential to establish a fair and universal measure against AMR that commits to ensuring all patients diagnosed with this infection have access to effective, safe, and narrow-spectrum antituberculosis treatments, along with measures to reduce inappropriate prescribing across public, private, and informal health systems [42,49,50,51,52].

All the above should strengthen intersectoral measures among the factors involved in the selection and spread of AMR under the One Health approach (human, animal, agricultural, and environmental health) [2,53]. This approach is relevant because the potential transmission of *M. tuberculosis* to other non-human species, as evidenced by studies in India and China, could pose a challenge in countries with high disease prevalence [54,55,56] with the risk of zoonotic transmission of resistant strains [49]. Moreover, environmental pollution and the inappropriate use of antibiotics in veterinary and agricultural settings could contribute to the increase in drug-resistant tuberculosis. Therefore, it is crucial to emphasize the proper management and disposal of antituberculosis drugs [57,58,59,60].

Among the limitations of this study, the researchers highlight the lack of molecular data; however, including phenotypic patterns allows identification of relevant trends and supports decision-making. Therefore, comparison with national INS reports and the guidelines of the National AMR Response Plan reinforces the usefulness of this analysis type for adapting local interventions, such as the effective implementation of ASPs and epidemiological surveillance based on standardized data from WHONET [7,8].

Additionally, the prediction intervals for the projected cases of the drug-resistant tuberculosis model in Cali were relatively broad, reflecting substantial uncertainty about future case counts; however, this is common in epidemiological time series characterized by high variability. Despite this limitation, the model does not underestimate the uncertainty, as it offers a realistic range of plausible future scenarios. Therefore, these intervals should be interpreted as plausible limits rather than precise predictions for decision-making. Another limitation could be the lack of clinical data, possible overestimation of resistance from a selection bias for patients’ severity, the acquisition of the infection (community or hospital), patient history, microorganism isolated, or therapies, that could be useful to analyze in the future.

Another limitation observed was that the models for the probability of hospitalization and death in patients with drug-resistant tuberculosis in Cali showed a relatively low pseudo-R^2^. However, in logistic regression models, pseudo-R^2^ values are generally low. The low pseudo-R^2^ suggests that the outcome is influenced by additional factors not included in the model. Nevertheless, odds ratios remain valid measures of association between exposure and outcome.

## 5. Conclusions

Antimicrobial resistance in Santiago de Cali is part of a broader health crisis, not an isolated issue, and requires comprehensive, sustainable, and evidence-based solutions. The findings highlight the urgent need for collaboration among the health sector, academia, and local authorities to enhance microbiological surveillance systems and develop strategies to improve antibiotic use.

In the Santiago de Cali District, where carbapenemases are endemic, this study underscores the importance of molecular investigations to complement phenotypic data in tracking the evolution of antimicrobial resistance. It also recommends implementing district-level guidelines that include molecular testing to detect coproductions and clinically relevant resistance patterns, thereby strengthening surveillance and supporting public health decision-making. Patients with drug-resistant tuberculosis who are also HIV-positive or homeless are more likely to be hospitalized or die. To reduce fatal outcomes from drug-resistant tuberculosis, it is crucial to prioritize public policies that ensure equitable, rapid, and timely access to more accurate diagnostic tests, along with initiatives to improve treatment adherence.

## Figures and Tables

**Table 1:** Distribution of isolates by body site and year, Cali, 2019–2024.

Body Site/Year	2019 *n* (%)	2020 *n* (%)	2021 *n* (%)	2022 *n* (%)	2023 *n* (%)	2024 *n* (%)	Total *n* (%)
**Blood (Blood Cultures)**	20,485 (20.0%)	176,153 (21.0%)	21,231 (20.0%)	7527 (18.0%)	17,898 (20.0%)	23,739 (21.0%)	267,033 (20.7%)
**Urinary tract (Urine)**	35,849 (35.0%)	285,201 (34.0%)	36,104 (34.0%)	15,472 (37.0%)	31,321 (35.0%)	38,434 (34.0%)	442,381 (34.2%)
**Respiratory tract**	18,436 (18.0%)	150,989 (18.0%)	19,108 (18.0%)	6273 (15.0%)	16,108 (18.0%)	20,348 (18.0%)	231,262 (17.9%)
**Surgical wound/soft tissue**	10,243 (10.0%)	75,494 (9.0%)	10,616 (10.0%)	4182 (10.0%)	8949 (10.0%)	11,304 (10.0%)	120,788 (9.4%)
**Invasive devices**	12,291 (12.0%)	117,436 (14.0%)	12,738 (12.0%)	6273 (15.0%)	10,739 (12.0%)	14,130 (12.5%)	173,607 (13.4%)
**Other sites**	5123 (5.0%)	33,553 (4.0%)	6359 (6.0%)	2091 (5.0%)	4473 (5.0%)	5088 (4.5%)	56,687 (4.4%)
**Total per year**	**102,427 (100%)**	**838,826 (100%)**	**106,156 (100%)**	**41,818 (100%)**	**89,488 (100%)**	**113,043 (100%)**	**1,291,758 (100%)**

n (%) of the annual total, Source: WHONET Surveillance, Santiago de Cali, cut-off date: 2024.

**Table 2:** Characteristics of the population diagnosed with drug-resistant tuberculosis in Cali, 2015–2024.

Variable		*N* (419)	Percentage (%)
Sex	Men	144	34.4
	Women	275	65.6
Life course	Childhood (0–11 years)	5	1.2
	Adolescence (12–17 years)	4	1.0
	Young adulthood (18–28 years)	97	23.2
	Adulthood (29–59 years)	227	54.2
	Older adulthood (60 and over)	86	20.5
Socioeconomic level	Low	203	48.4
	Middle	59	14.1
	High	10	2.4
	No Data	147	35.1
Type of social security	Contributive Regime	142	33.9
	Subsidized Regime	217	51.8
	Special Regimes	10	2.4
	Exception category	11	2.6
	Uninsured	36	8.6
	Undefined status	3	0.7
Resistance type	Extensively resistant (XDR)	3	0.7
	Monoresistant	184	43.9
	Multidrug-resistant (MDR)	123	29.4
	Pan-resistant (PDR)	4	1.0
	Pre-XDR	1	0.2
	Rifampicin resistant	91	21.7
	Under classification	13	3.1

Source: SIVIGILA, cut-off date 4 April 2025.

**Table 3:** Probability of hospitalization and death among patients with drug-resistant tuberculosis in Cali, 2015–2024.

Variable (*n* = 419)	OR Hospitalization	95% IC	*p* Value	OR Death	95% IC	*p* Value
VIH	**5.597**	3.098; 10.111	**0.000**	**3.340**	1.717; 6.491	**0.000**
Homeless	**2.938**	1.486; 5.811	**0.002**	**2.596**	1.162; 5.791	**0.020**

Source: SIVIGILA, cut-off date 4 April 2025.

## Data Availability

Since 2016, the Health Secretariat of the Special District of Santiago de Cali has published fifty-six open data sets on the portal www.datos.cali.gov.co, which serves as the primary source of information analyzed in this study. Available in https://www.cali.gov.co/salud/publicaciones/139966/datos-abiertos/ (accessed on 15 May 2025).
